# Investigating various nonlinear vibration problems using VIBRANT: A tool based on Abaqus and Python

**DOI:** 10.1371/journal.pone.0338419

**Published:** 2026-01-21

**Authors:** Mertol Tüfekci, Furkan Sevencan, Ozan Yurdakul

**Affiliations:** 1 Centre for Engineering Research, University of Hertfordshire, Hatfield, United Kingdom; 2 School of Physics, Engineering and Computer Science, University of Hertfordshire, Hatfield, United Kingdom; 3 TRMotor Propulsion Systems Inc., Hacettepe Technopolis, Ankara, Turkey; IGDTUW: Indira Gandhi Delhi Technical University for Women, INDIA

## Abstract

This paper investigates nonlinear vibration problems using VIBRANT (VIbration BehaviouR ANalysis Tool), a tool based on Python and Abaqus for the detailed analysis of complex mechanical systems. VIBRANT employs time-marching algorithms to perform time domain finite element simulations under harmonic excitation, predicting frequency domain behaviour. It addresses a significantly large range of nonlinearities, including contacts and large displacements, as it uses a commercial finite element software package Abaqus, while reducing computational time through parallelisation. The tool’s capabilities are examined through three academic benchmark examples. The first example examines a geometrically nonlinear Timoshenko beam subjected to large displacements, which highlights the nonlinear behaviour due to significant deformation associated with stiffness nonlinearities. The second example is a bar forced to move in axial direction by its frictional clamps that are modelled using Jenkins contact elements. This example is also a demonstration of a stiffness nonlinearity. The third example involves an Euler-Bernoulli beam with a frictional contact element, which demonstrates the effects of damping nonlinearities by the application of a localised Coulomb friction element. All examples serve to validate VIBRANT’s accuracy and efficiency in capturing the characteristics of nonlinear systems, emphasising its potential for industrial applications, particularly in aerospace engineering. VIBRANT’s capacity to model a wide range of nonlinearities and to automate frequency sweep analysis with minimal manual intervention represents a significant advantage, providing a reliable and efficient approach to modelling and analysing dynamic responses in engineering structures.

## 1 Introduction

The analysis of nonlinear mechanical systems poses significant challenges in engineering, particularly for systems undergoing large deflections, experiencing contact interactions, and influenced by nonlinear material properties [1–5]. Complex systems, such as those found in various engineering fields, require sophisticated analysis tools to capture their dynamic behaviour accurately. Among these systems, the dynamics of aeroengine components and assemblies stand out due to their complexity and critical performance requirements [6–8]. These components, particularly bladed disc systems, must operate under extreme conditions, making their accurate modelling essential to ensure reliability and performance [9].

Nonlinear effects play a decisive role in the dynamic behaviour of aeroengine assemblies. Friction at contact interfaces, partial slip at under-platform dampers, and large-amplitude motions of rotating blades introduce amplitude-dependent stiffness and energy dissipation that cannot be captured by linear models. These nonlinearities are responsible for the shift of natural frequencies, generation of superharmonics, and complex hysteretic damping observed in experimental tests. Comparable nonlinear phenomena are also reported in geared or locally resonant structures, where bifurcations and amplitude-dependent bandgap shifts emerge under harmonic excitation [10–12]. Therefore, accurate prediction of nonlinear dynamic responses is crucial for ensuring the structural integrity and lifing assessment of engine components.

Under such extreme thermal and centrifugal environments, components experience significant interface friction, contact separation, and joint preload variation. Even apparently simple sub-assemblies such as bolted or frictional joints display path-dependent stiffness and damping that remain open challenges in high-fidelity modal analysis. Recent developments in nonlinear continuation and model-reduction frameworks show that addressing these challenges requires coupling frequency- and time domain formulations capable of capturing localised nonlinearities and stochastic effects [13,14].

Nonlinear mechanical vibration modelling techniques employ a variety of methods to analyse and predict the behaviour of systems under different conditions. Two prominent methods are the Harmonic Balance Method (HBM) and time-marching techniques. HBM is a frequency domain technique that approximates the solution of nonlinear differential equations as a sum of harmonics, transforming time-dependent problems into algebraic equations for computational efficiency in periodic solutions. This method is particularly effective for large-scale systems [15], unsteady nonlinear behaviours [16], and stochastic analysis under uncertain conditions [17]. Additionally, HBM facilitates model order reduction in nonlinear systems, which is crucial for managing complex geometrical nonlinearities [18].

HBM has been widely applied to study the nonlinear mechanical vibrations of systems influenced by friction, providing a means to model dampers’ nonlinear behaviour effectively [16,19–21]. Additionally, time-marching methods have demonstrated efficacy in capturing nonlinear mechanical vibrations, with good agreement between solutions and experimental data revealing intricate dynamics within friction brake systems [22–25].

Several adaptations and improvements to HBM have been developed. The Receptance Harmonic Balance Method (RHBM) is effective for aero-engine models with nonlinear bearings, offering significant computational efficiency when combined with time-marching solvers [26]. The Global Residue Harmonic Balance Method (GRHBM) enhances accuracy by incorporating all former global residual errors, making it particularly useful for analysing nonlinear vibrating beams [27]. The Alternating Frequency/Time (AFT) domain technique addresses nonlinearities in fractional exponential models, handling complex nonlinear problems efficiently [28–32].

Time-marching methods are time domain techniques that solve problems step-by-step through time, providing versatility but often at a higher computational cost for long-term integrations. These methods are widely applicable to various problems, including stiff systems [33,34], and can be improved using pseudotime marching with local time stepping and multigrid acceleration for enhanced efficiency [16]. Advanced schemes in time marching ensure numerical stability, especially for systems with stiff equations [33].

Lacayo et al. underline the necessity of both frequency and time domain simulations for correct analysis of large nonlinear systems, emphasising that a mixture of both domains is required for thorough knowledge and dependable modelling [35]. For the purpose of comprehending the nonlinear vibrations of systems such as turbine blade-disc systems with underplatform dampers, precise modelling and analytical approaches are necessary. Crucial elements in these studies are the precision of contact pressure distribution and the taking into account of zero-harmonic terms in multiharmonic expansion [36]. Dynamic responses and variations in natural frequency can be accurately predicted by theoretical models that incorporate empirically determined contact parameters [37]. The integration of dry friction dampers into turbine blade studies has been made easier by quasi-linearisation techniques, which convert nonlinear differential equations into more understandable algebraic forms [38].

Numerous computer techniques and tools have been developed recently to address the difficulties associated with nonlinear system analysis. NLvib is notably adept in examining nonlinear structures, such contacts, which are especially pertinent to aeroengine turbines because of their influence on structural integrity and performance [16]. The French-developed MANLAB is a comprehensive tool for the continuation of periodic solutions in nonlinear systems. It effectively explores the solution space of nonlinear equations by combining the asymptotic numerical method with the harmonic balance method [39–41]. Although it does not have continuation capabilities, another important tool is the open-source HB solver Mousai, which offers a general-purpose solution to nonlinear vibration problems [42].

FORSE, an in-house code developed at Imperial College London, is recognised for its powerful capabilities in modelling the frequency domain forced response of nonlinear systems [43–48]. Additionally, PERMAS, a software package developed by Intes, performs harmonic balance on nonlinear systems to predict their dynamic behaviour [49,50]. The combined use of these advanced computational tools underscores the importance of leveraging both frequency and time domain simulations to achieve accurate and reliable modelling of complex nonlinear systems.

The most recent nonlinear vibration research increasingly focuses on integrating continuation-based bifurcation tracking with reduced-order and frequency–time hybrid methods. Approaches such as the nonlinear Wave Finite Element Method and multi-parametric optimisation frameworks have demonstrated that nonlinearities, when properly resolved, can drastically alter resonance placement and stability characteristics [11,12]. These studies collectively emphasise that a practical computational environment must not only capture complex nonlinear effects but also remain compatible with industrial-scale finite element workflows—precisely the gap that VIBRANT addresses.

The aim of this study is to investigate nonlinear vibration problems by using VIBRANT on representative academic benchmark examples. Conventional methods of frequency sweep analysis often have a restricted range of nonlinearities that they can analyse. Here, VIBRANT is employed to study the dynamic behaviour of complex mechanical systems through time-marching simulations. VIBRANT is a time-marching approach for high-fidelity time domain simulations that predicts frequency domain behaviour under harmonic stimulation. It is developed with Abaqus and Python. It can handle a wide range of systems modelled in Abaqus and minimise computing time through its ability to parallelise calculations. Three examples that deal with damping nonlinearities and stiffness are considered to illustrate VIBRANT’s application. In the first case, stiffness nonlinearities are highlighted by examining a geometrically nonlinear Timoshenko beam enduring substantial displacements. In the second example, which also demonstrates stiffness nonlinearity, a bar is subjected to axial motion by its frictional clamps and is modelled using Jenkins contact elements. In the third example, a localised Coulomb friction element is applied to an Euler-Bernoulli beam with a frictional contact element to illustrate damping nonlinearities. When a steady state is attained, VIBRANT applies harmonic excitation and tracks reactions methodically, monitoring vital characteristics such as vibration damping, amplitude, and kinetic and potential energies throughout an appropriate frequency range. These examples demonstrate how VIBRANT can be used in academic studies of nonlinear vibration, while also highlighting its potential to support future design and analysis of engineering systems, where parametric studies can investigate the impact of design parameters on dynamic behaviour [34,51,52].

## 2 Overview of VIBRANT

VIBRANT (VIbration BehaviouR ANalysis Tool) is a computer program that is designed to automate and perform frequency sweep analysis of generic mechanical systems by integrating the robust capabilities of Abaqus with Python’s flexibility when it comes to scripting. VIBRANT performs accurate time domain simulations, producing frequency domain results and handling a wide range of nonlinearities and multiphysical phenomena. It is particularly effective in scenarios involving nonlinearities like contact, local plastic deformations, and damping characteristics. Despite its computational intensity, VIBRANT mitigates load through parallelisation, making it efficient and user-friendly for modern mechanical and aerospace engineers.

### 2.1 Software dependencies

VIBRANT leverages several key components: Abaqus and Python are used for model setup, simulation, and data extraction. **NumPy** is employed for numerical operations, while **Matplotlib** is utilised for data visualisation. Additionally, standard Python libraries such as *os*, *math*, and *time* are incorporated for system operations.

To use VIBRANT, place the Python script in the same directory as the Abaqus files or specify its path. Ensure both Abaqus and Python are correctly installed and configured to avoid compatibility issues.

### 2.2 Architecture and workflow

**Fig 1:**
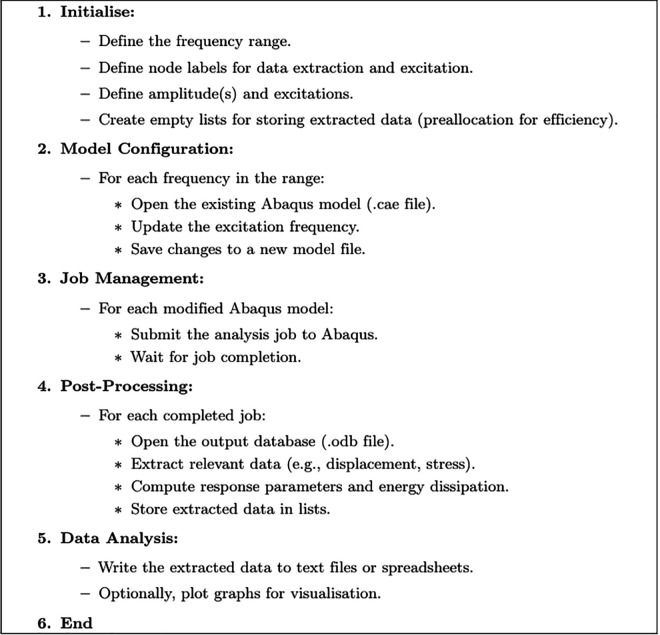
Outline of the framework that the software follows.

VIBRANT is focused on dynamic analysis within Abaqus through its several key modules, each performing specific tasks as shown in Fig 1.

## 3 Numerical examples

This section illustrates three numerical examples that validate and show the capabilities of VIBRANT. For comparative purposes, two examples are also solved using NLvib, a well-established numerical tool written in MATLAB that utilises HBM and continuation, a fundamentally different approach from VIBRANT [53–57]. The other example compares VIBRANT’s performance with an example available in the literature, featuring both an analytical solution and a numerical solution performed with HBM [52].

### 3.1 Numerical implementation details

In all examples, the structures are subjected to harmonic forcing of the form $F_{ex}(t)=F_{0}\sin(\Omega t)$, and frequency response curves (FRFs) are obtained by sweeping the excitation frequency across a range that captures the first resonance. The FRF is constructed using the steady-state root-mean-square (RMS) displacement response, extracted after the decay of transients. For consistency of comparison, the excitation frequency is normalised by the first natural frequency of the corresponding linear system, and the response amplitudes are normalised by the linear steady-state displacement at resonance.

Time domain simulations in VIBRANT are performed through Abaqus with geometric nonlinearity enabled (NLGEOM=ON). The nonlinear restoring force is evaluated at each time step, and the resulting displacement histories are used to compute the FRFs. In contrast, NLvib employs the Harmonic Balance Method (HBM) and continuation to directly compute periodic steady-state solutions in the frequency domain. These two fundamentally different approaches allow cross-validation of the nonlinear response predictions and provide confidence in the robustness of the numerical results.

### 3.2 Example 1: Timoshenko beam undergoing large displacements

The first example involves a Timoshenko beam subjected to large displacements, illustrating VIBRANT’s capability to handle geometric nonlinearities, which is a form of distributed nonlinearity. Both VIBRANT and NLvib are utilised to perform the simulation, and their results are compared to ensure consistency and accuracy in capturing the beam’s dynamic behaviour under forcing that leads to large displacement.

The geometrically nonlinear Timoshenko beam employed in this study is discretised into 21 elements. The properties of the model are taken from the publicly available example in NLvib’s repository [57,58]. Ten harmonics are used when the beam is modelled using NLvib. The beam dimensions are set to a height (*h*) of 40 mm, a width (*b*) of 40 mm, and a length (*L*) of 1200 mm. The Young’s modulus (*E*) is 90 GPa, the shear modulus (*G*) is calculated as:

$$G=\frac{E}{2(1+\nu )}$$ (1)

where ν is Poisson’s ratio. The density (*ρ*) is $7850\times 10^{-9}$ kg/mm^3^. The damping ratio is denoted by ξ, with the viscoelastic constants for axial (*μ*) and shear deformation (*γ*) determined by:

$$\mu =\xi \times 2\times \sqrt{E\times \rho \times h\times b}$$ (2)

$$\gamma =\xi \times 2\times \sqrt{G\times \rho \times h\times b}$$ (3)

**Fig 2:**
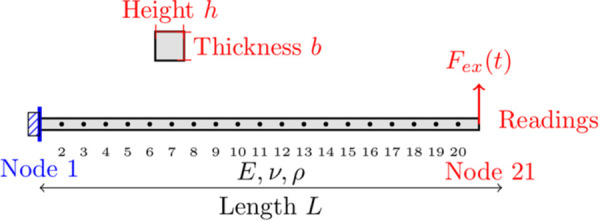
Schematic visualisation of the 21-node Euler-Bernoulli beam with excitation at the free tip. Readings are taken from Node 21.

The system, as shown in Fig 2, is excited at its tip with harmonic forces of varying magnitudes to explore its nonlinear response. This example allows the study of the beam’s frequency response and validates the performance of the VIBRANT software against the established NLvib using HBM. By comparing these two methods and software packages, the computational accuracy and robustness of VIBRANT are demonstrated, showing its ability to handle such nonlinear systems accurately which are common in many mechanical and aerospace engineering applications. Some examples could be aircraft engine fan blades, helicopter blades, and aircraft wings [6,7,59].

To emphasise, the only source of nonlinearity in this example is geometric, arising from large displacements and associated nonlinear strain–displacement coupling in the Timoshenko beam formulation. No local nonlinearities are introduced. In VIBRANT, this behaviour is captured via Abaqus by activating the NLGEOM=ON option, which renders the stiffness displacement-dependent, *K*(*u*), resulting in a distributed geometric nonlinearity. This allows the method to capture stiffening effects that occur under large deformation.

### 3.3 Example 2: Axial vibration of a bar with frictional clamps

**Fig 3:**
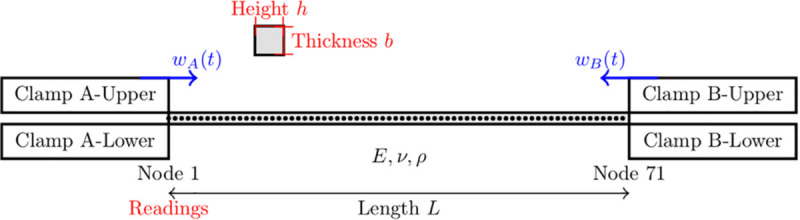
Schematic visualisation of the bar system with clamps and excitation forces. Readings are taken from Node 71.

As the second example, forced axial vibrations of a bar with frictional clamps are investigated. For the axial vibration study, the bar dimensions are as follows: a length of 70 mm, a width of 14 mm, and a thickness of 4 mm. The rod is made of a material with a density of 1400 *kg*/*m*^3^, a Young’s modulus of 3.5 GPa, and a Poisson’s ratio of 0.38. The model uses a one-dimensional finite element bar element with 71 nodes, each having a single degree of freedom to capture the bar’s axial motion. To represent the clamps, a Jenkins contact element is attached to the nodes that are at the tip. This example is taken from the literature where an analytical and a numerical solution employing AFT HBM are presented providing a different validation for VIBRANT against an analytical solution and a different computer program that uses HBM other than NLvib [52]. This example addresses a different type of nonlinearity that is localised and related to the stiffness. The investigated system is shown in Fig 3.

The frictional clamps introduce a localised nonlinearity, modelled using a Jenkins element with an elastic stick regime and a slip force limited by the classical Coulomb threshold, $\mu F_{N}$. The elastic stage is governed by a spring of stiffness *k*_*s*_, after which sliding occurs once the restoring force reaches $\mu F_{N}$. The values of *μ* and *F*_*N*_ are taken directly from the reference [52]. The nonlinear elements are attached at the axial DOFs of Nodes 1 and 71, allowing the stick–slip transitions to govern the axial vibration response of the rod.

### 3.4 Example 3: Cantilever beam with contact at the tip

This third example examines a cantilever Euler-Bernoulli beam excited by a concentrated force applied at its midpoint (Node 11). The beam is modelled using a one-dimensional finite element approach and a nonlinear dry (Coulomb) friction element attached to its free end (Node 20). The friction force is dependent on the velocity of the contact point, $\dot{x}$, utilising the $\tanh(\dot{x})$ function as an approximation of the nonlinearity, simulating a localised interaction between the beam and an external component. Such scenarios are common in practical engineering systems where contact dynamics play a critical role [60]. The system’s dynamic response is analysed and compared using both VIBRANT and NLvib to validate the accuracy and effectiveness of VIBRANT.

The Euler-Bernoulli beam is characterised by a length (*L*) of 2 m, a height (*h*) of 0.1 m, a thickness (*b*) of 0.15 m, a Young’s modulus (*E*) of 185 GPa, and a density (*ρ*) of 7830 kg/m^3^. The beam is discretised into 19 finite elements, creating a model with 20 nodes. The first node is clamped, representing a fixed boundary condition, while the twentieth node is free and subjected to an excitation force *F*_*ex*_. A nonlinear dry friction element is introduced at the fourth node, which utilises the $\tanh(\dot{z}\;t)$ function to simulate frictional behaviour, where *z* is the spatial coordinate aligned with the transverse deflection of the beam, $\dot{z}$ denotes the first time derivative of *z*, and *t* represents time.

**Fig 4:**
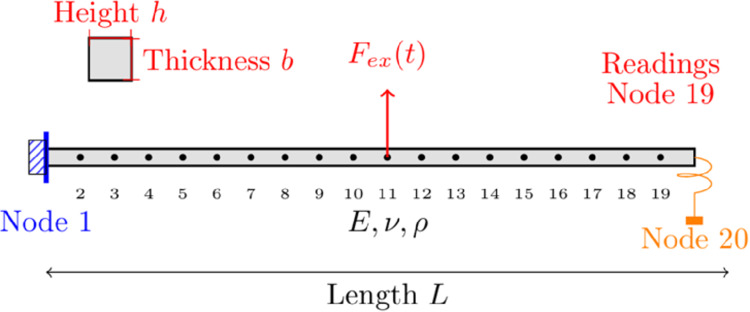
Schematic visualisation of the 20-node Euler-Bernoulli beam with excitation from the middle and friction contact element at the free tip. Readings are taken from Node 19.

The system configuration is illustrated in Fig 4. The dynamic response of the beam, particularly focusing on the first bending mode, is analysed over a frequency range that encompasses the first peak in the frequency response curves. The presence of the frictional contact element is expected to alter the system’s response, maintaining the first peak within the bounds established by linear cases without the contact element, and with the tip node fixed, where the contact element is initially attached.

The system is analysed using two different computational tools: the MATLAB toolbox NLvib and VIBRANT. NLvib specialises in the analysis of nonlinear vibrations using the HB method, enabling efficient computation of periodic solutions. VIBRANT, on the other hand, is designed for the analysis of vibrations in complex mechanical systems through Abaqus, using time domain simulations and time-marching techniques.

The comparison involves evaluating the system’s response under different maximum friction forces, $\mu F_{N}$, where *μ* is the friction coefficient and *F*_*N*_ is the normal contact force. This analysis highlights VIBRANT’s robustness and precision in addressing damping nonlinearities within complex mechanical systems.

## 4 Results and discussions of the examples

### 4.1 Example 1: Timoshenko beam undergoing large displacements

**Fig 5:**
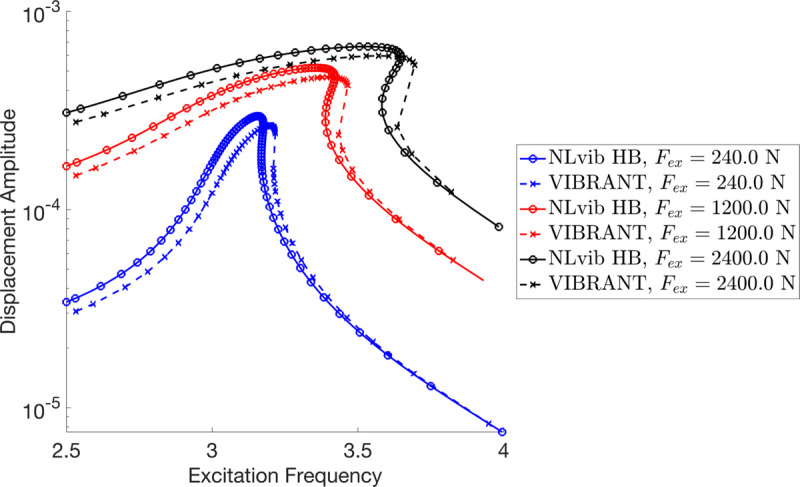
Frequency response of the cantilever Timoshenko beam subjected to large displacements, showing displacement amplitudes for different excitation force levels (Readings are taken from the free tip and units in rad/s for frequency and m for displacement).

Fig 5 shows the frequency response of a geometrically nonlinear Timoshenko beam subjected to large displacements. The responses were obtained using both VIBRANT and NLvib for three different excitation force levels: $F_{ex}=240.0\;\text{N}$, 1,200.0N, and 2,400.0N. The displacement amplitude is plotted against the excitation frequency on a logarithmic scale.

The figure illustrates that both VIBRANT and NLvib show consistent results across the range of excitation forces, demonstrating good agreement between the two methods. As the excitation force increases, the displacement amplitude also increases, and the peak amplitude shifts to higher frequencies, indicating a stiffening nonlinearity due to large displacements. This shift is more pronounced at higher excitation levels.

For $F_{ex}=240.0\;\text{N}$ (blue lines), the frequency response shows a clear resonance peak around 3.1 Hz. The resonance peak remains well-defined and consistent between both VIBRANT and NLvib, with only minor differences in the displacement amplitude.

At $F_{ex}=1,200.0\;\text{N}$ (red lines), the resonance peak shifts slightly to the right, occurring at around 3.2 Hz. The displacement amplitudes for this excitation level are higher, as expected, and again, VIBRANT and NLvib results are in good agreement, with VIBRANT showing a slightly lower peak amplitude.

For the highest excitation force, $F_{ex}=2,400.0\;\text{N}$ (black lines), the resonance peak further shifts to approximately 3.3 Hz. The results from both methods show increased displacement amplitudes, with VIBRANT again slightly underestimating the peak amplitude compared to NLvib. This trend suggests that VIBRANT may have a slightly conservative approach in predicting peak displacements for very high excitation forces.

### 4.2 Example 2: Axial vibration of a bar with frictional clamps

**Fig 6:**
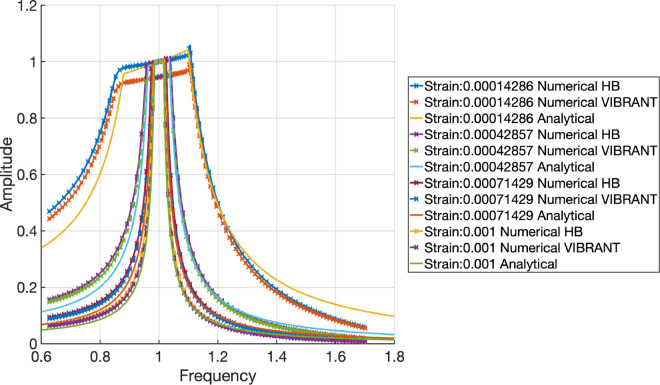
Frequency response of the bar with frictional clamps for different levels of imposed strain (Readings are taken at the contact point of the beam and the clamps and units are nondimensionalised for frequency by dividing the frequency axis by the reference value of the first axial mode of a clamped-clamped beam and displacement by dividing the displacement amplitude values by the displacement amplitude imposed on the clamps).

Fig 6 shows the frequency response of the forced axial vibrations of a bar with frictional clamps for different levels of imposed displacement/strain (strain is defined as imposed displacement divided by the length of the bar *w*_*A*_/*L*) levels of 0.00014286, 0.00042857, 0.00071429, and 0.001 on the clamps. The results obtained from numerical solutions performed by the mentioned HB code, VIBRANT, and analytical solutions, are presented in nondimensional form, with the frequency axis divided by the reference value of the first axial mode of a clamped-clamped beam and the displacement amplitude values divided by the displacement amplitude imposed on the clamps.

A key observation from the results is the presence of flat tops in the amplitude responses, indicative of full stick behaviour where there is no slip. This occurs when the entire frictional contact is engaged, and the bar moves without relative displacement at the frictional interface. At lower strain levels, the responses exhibit this flat-top characteristic, implying full stick behaviour predominates.

As the strain level increases, the amplitude starts to deviate from the flat-top, indicating the onset of slip behaviour. The analytical solutions, Numerical HB, and Numerical VIBRANT results align closely, validating the performance of VIBRANT in capturing the dynamics of the bar with frictional clamps. The minor deviations seen in the results can be attributed to the intrinsic damping present in the Abaqus model used by VIBRANT. This intrinsic damping prevents the amplitude from reaching the theoretical maximum value of 1, even under full stick conditions.

The consistent flat tops across various strain levels in the Numerical VIBRANT results confirm that VIBRANT accurately models the stick-slip transition. The slight reduction in amplitude due to intrinsic damping does not significantly impact the overall trend, which still reflects the expected dynamic behaviour of the system.

With reference to Fig 6, it can be observed that when the normal load is constant, the full-stick regime dominance within the selected frequency interval shrinks when the clamp motion amplitude, or *W*_*A*_/*L* ratio, increases. This means that the slip region eventually expands and increases friction damping. The highest amplitude is found beyond the clamp motion amplitude near the conclusion of the post-resonance full-stick regime. The existence of contact stiffness, which also regulates the stick regime’s slope, provides an explanation for this. The system is not immediately activated by a simple harmonic force, and the stiffness of the bar is not the sole factor contributing to the system’s overall stiffness. In this instance, a Jenkins element describes the interaction between the moving clamp and the bar over the contact. As a result, the bar’s amplitude can somewhat exceed the clamp motion’s amplitude. For the same reason, there is a little rise near the end of the stick region.

### 4.3 Example 3: Cantilever beam with contact at the tip

**Fig 7:**
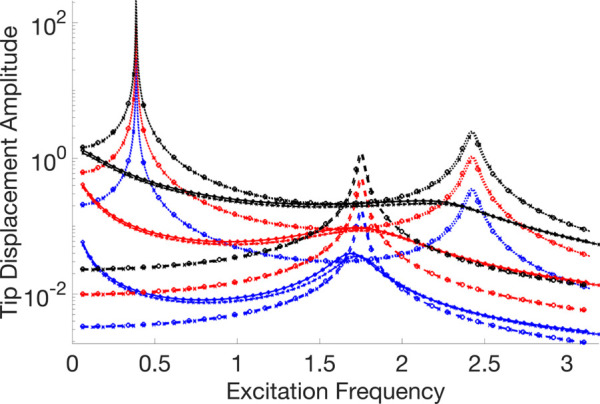
Frequency response curves of the cantilever beam with a localised frictional contact at its free tip for $\mu F_{N}=3.00$. The nonlinear contact element is applied at Node 20, producing stick–slip behaviour that alters the dynamic response relative to the linear reference case.

**Fig 8:**
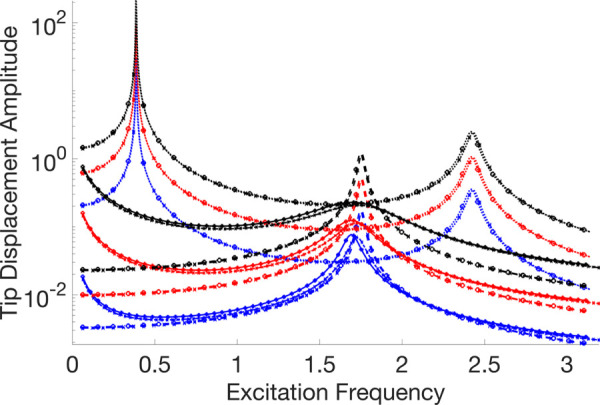
Frequency response curves of the cantilever beam with a localised frictional contact at its free tip for $\mu F_{N}=10.00$. The nonlinear contact element is applied at Node 20, producing stick–slip behaviour that alters the dynamic response relative to the linear reference case.

**Fig 9:**
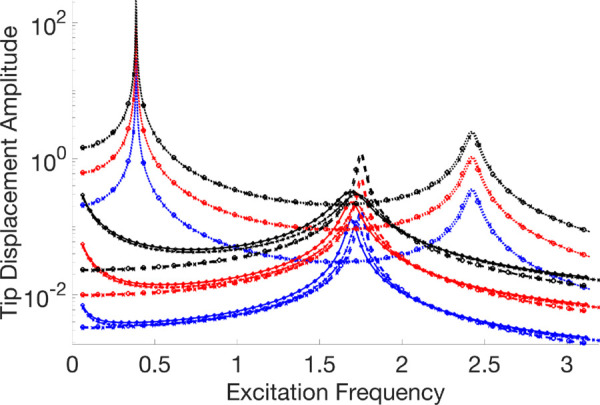
Frequency response curves of the cantilever beam with a localised frictional contact at its free tip for $\mu F_{N}=30.00$. The nonlinear contact element is applied at Node 20, producing stick–slip behaviour that alters the dynamic response relative to the linear reference case.

**Fig 10:**
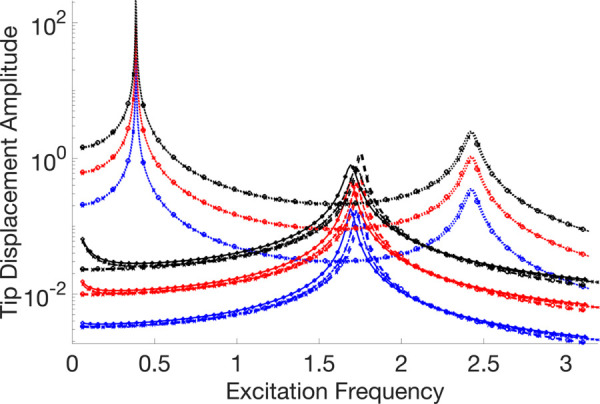
Frequency response curves of the cantilever beam with a localised frictional contact at its free tip for $\mu F_{N}=150.00$. The nonlinear contact element is applied at Node 20, producing stick–slip behaviour that alters the dynamic response relative to the linear reference case.

**Fig 11:**
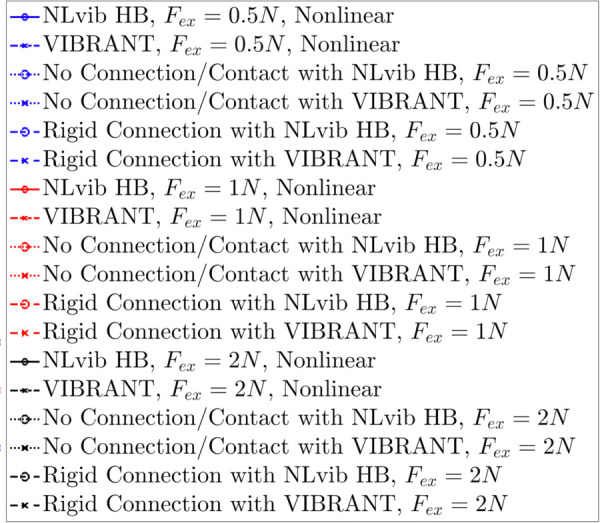
Legend for the frequency response curve figures showing the different response types and analysis methods used in the cantilever beam with frictional contact study.

A thorough visual comparison between the linear (with fixed contact and without any contact) and nonlinear dynamic responses of an Euler-Bernoulli beam subject to harmonic stimulation is provided by the frequency response curves displayed in Figs 7–11. All of these examples’ computations are carried out using VIBRANT and NLvib. The frequency response, *F*_*ex*_, as determined by NLvib and VIBRANT for various excitation amplitudes, is shown by the curves in each panel. Plots show how the system behaves as it transitions from a dynamic regime that is linear to one that is heavily influenced by frictional contact that is not linear.

The contact forces and the characteristics of the Euler-Bernoulli beam, which has a localised frictional contact at the tip, have a major impact on its dynamic behaviour. The coefficient of friction, *μ*, and the amplitude of excitation, *F*_*ex*_, are the main factors that govern this interaction. They determine the stick, slip, and stick-slip behaviours, respectively, where the surfaces slide against one another, move together without relative motion, and both are observed during the motion.

The system first shows a stiffening effect as *μ* grows because stick behaviour predominates, which resists motion and enhances the structure’s apparent stiffness. This behaviour is intricate and heavily reliant on the frictional contact properties and vibration amplitude.

The beam mainly stays in the stick phase at smaller excitation amplitudes, indicating that the nonlinearity-induced stiffness change is negligible. However, the system enters the slip phase more frequently as the excitation force increases, especially at peak vibration velocities. Through frictional damping, energy is dissipated during this transition, resulting in a softening effect that is typified by smaller peak amplitudes in the frequency response. It is not enough to say that the nonlinear response shows an increase in stiffness. Rather, it depicts the energy loss at the frictional contact.

As a result, the nonlinear frictional damping is essential to how the system reacts to stronger excitation forces. It stops the peak amplitudes from increasing linearly, which is what one would anticipate in a frictionless system. As a result, there is no proportional connection between *F*_*ex*_ and peak amplitudes in the frequency response curves that arise. Rather, they disclose a complicated reliance on both *μ* and *F*_*ex*_, which controls the dynamic shift between stick and slip regimes and forms the nonlinear behaviour that is seen.

The dynamic equilibrium between inertia, stiffness, and damping inside the beam structure governs the physics underlying these results. Additional forces that depend on displacement and velocity are introduced by the insertion of a frictional contact element at the tip; these forces do not exist in a linear setting. The system’s equilibrium is changed by these forces, which become more noticeable as the excitation frequency gets closer to the natural frequency. This leads to the observed departure from linear behaviour.

It is evident that the outcomes generated by NLvib and VIBRANT exhibit a high degree of agreement. The two sets of curves show good agreement, however the amplitude computed using VIBRANT appears to be consistently less than the amplitude computed using NLvib. This difference may be explained by the artificial/extra damping that NLvib does not include, but Abaqus provides by default in its element definition in order to aid numerical convergence and stability in time domain simulations. As a result, it is discovered that the amplitudes computed using VIBRANT are about up to 11% (maximum observed error) lower than those obtained using NLvib.

## 5 Conclusion

The purpose of VIBRANT: VIbration BehaviouR ANalysis Tool, which is presented in this paper, is to improve computational modelling of complicated mechanical systems, especially in the field of aeronautical engineering. VIBRANT combines the scripting flexibility of Python with the robustness of Abaqus to enable accurate, high-fidelity time domain simulations that transfer into frequency domain analysis. Because of VIBRANT’s ability to compute in parallel, considerably less computational time is required to handle a variety of systems and nonlinearities that are simulated using Abaqus.

The tool mainly serves the domains of mechanical and aeronautical engineering by offering important insights into intricate nonlinear systems, which may have an impact on the advancement of aircraft engines in the future. When it comes to investigating dynamic systems and laying the groundwork for further studies and practical applications, VIBRANT is a strong and trustworthy instrument. The creation and use of the tool in this work emphasises the advantages of integrating robust time domain solvers for complicated systems and advances computational modelling.

Three numerical examples demonstrate VIBRANT’s capabilities and validate its performance. The first example, involving a geometrically nonlinear Timoshenko beam subjected to large displacements, highlighted VIBRANT’s proficiency in capturing geometric nonlinearities and provided a comparative analysis against NLvib. The second example explored forced axial vibrations of a bar with frictional clamps, revealing detailed dynamics and validating results against analytical solutions and NLvib. The third example, focusing on an Euler-Bernoulli beam with a frictional contact element at its tip, showcased VIBRANT’s effectiveness in handling damping nonlinearities and provided a comprehensive comparison with NLvib’s harmonic balance method.

Results consistently showed excellent agreement between VIBRANT and NLvib, demonstrating VIBRANT’s robustness and accuracy in modelling complex nonlinear systems. Minor deviations in amplitude responses, attributed to intrinsic damping in Abaqus, were within acceptable limits, underscoring VIBRANT’s reliability.

VIBRANT’s capability to model a wide range of nonlinearities and automate frequency sweep analysis with minimal manual intervention represents a significant advancement in the field of nonlinear vibration analysis. The tool’s proficiency in handling complex interactions within mechanical systems makes it particularly suitable for industrial applications, especially in aerospace engineering.

For future work, the addition of continuation methods could enable the capture of unstable branches in the frequency response, providing a more comprehensive understanding of the system’s dynamic behaviour. Implementing model order reduction techniques could further reduce computational load, making VIBRANT even more efficient and applicable to larger, more complex systems.

By addressing these areas, VIBRANT can further enhance its performance, offering more detailed insights into the dynamic behaviour of nonlinear systems and demonstrating its applicability across various engineering fields.
